# Are patients’ preferences regarding the place of treatment heard and addressed at the point of referral: an exploratory study based on observations of GP-patient consultations

**DOI:** 10.1186/1471-2296-14-189

**Published:** 2013-12-10

**Authors:** Aafke Victoor, Janneke Noordman, Johan A Sonderkamp, Diana M J Delnoij, Roland D Friele, Sandra van Dulmen, Jany J D J M Rademakers

**Affiliations:** 1 NIVEL, the Netherlands Institute for Health Services Research, P.O. Box 1568, 3500, BN Utrecht, the Netherlands; 2 Tilburg University, School of Social and Behavioural Sciences, Tranzo, P.O. Box 90153, 5000, LE Tilburg, the Netherlands; 3 Health Care Insurance Board (CVZ), Quality Institute, P.O. Box 320, 1110, AH Diemen, the Netherlands; 4 Department of Primary and Community Care, Radboud University Nijmegen Medical Centre, P.O. Box 9101, 6500, HB Nijmegen, the Netherlands; 5 Department of Health Science, Buskerud University College, Drammen, Norway

**Keywords:** Choice behaviour, Patient freedom of choice laws, Healthcare providers, Healthcare reform, Communication, General practitioners, Referral, Physicians’ role

## Abstract

**Background:**

Today, in several north-western European countries, patients are encouraged to choose, actively, a healthcare provider. However, patients often visit the provider that is recommended by their general practitioner (GP). The introduction of patient choice requires GPs to support patients to be involved, actively, in the choice of a healthcare provider. We aim to investigate whether policy on patient choice is reflected in practice, i.e. what the role of the patient is in their choices of healthcare providers at the point of referral and to what extent GPs’ and patients’ healthcare paths influence the role that patients play in the referral decision.

**Methods:**

In 2007–2008, we videotaped Dutch GP-patient consultations. For this study, we selected, at random, 72 videotaped consultations between 72 patients and 39 GPs in which the patient was referred to a healthcare provider. These were analysed using an observation protocol developed by the researchers.

**Results:**

The majority of the patients had little or no input into the choice of a healthcare provider at the point of referral by their GP. Their GPs did not support them in actively choosing a provider and the patients often agreed with the provider that the GP proposed. Patients who were referred for diagnostic purposes seem to have had even less input into their choice of a provider than patients who were referred for treatment.

**Conclusions:**

We found that the GP chooses a healthcare provider on behalf of the patient in most consultations, even though policy on patient choice expects from patients that they choose, actively, a provider. On the one hand, this could indicate that the policy needs adjustments. On the other hand, adjustments may be needed to practice. For instance, GPs could help patients to make an active choice of provider. However, certain patients prefer to let their GP decide as their agent. Even then, GPs need to know patients’ preferences, because in a principal-agent relationship, it is necessary that the agent is fully informed about the principal’s preferences.

## Background

Patient choice of healthcare providers is currently an important element of the healthcare systems of various north-western European countries and is often supported by law. The rationale for this is twofold. Firstly, it empowers patients and gives them the opportunity to influence their own care process. Secondly, choice was introduced as one element of regulated competition [1-3]. Within this context, patients are viewed as autonomous healthcare consumers [3] and are expected to make active choices or, put differently, deliberate choices between healthcare providers. These choices would, in theory, be based on comparing information on quality and price [4]. This should enhance competition between providers [2,3] and, ultimately, result in a more personalised, responsive, efficient, and higher quality health service [3,5]. This line of reasoning originates from the neoclassical microeconomic theory [6]. However, in practice, instead of making active choices, patients tend to visit the default provider, often simply the one that is recommended by their general practitioner (GP) [7-10].

The fact that GPs can play a major role in deciding where patients go for specialist care is partly because in several countries, such as the UK and the Netherlands, patients need a referral from a GP before they can access specialist care [11]. Additionally, patients may be reluctant to decide on a provider [3], because they do not always perceive differences in quality of providers [12], they often have no insight into the quality of providers [13,14], and are often unaware of the costs that they incurred because they commonly do not directly pay for services [4]. Instead they expect their GP to act as their agent and understand and use performance information when making referral decisions [9]. In a principal–agent relationship, the choice that is made by the agent is the one that the principal would have made if they were themselves fully informed. It is therefore necessary that the agent is fully informed about the principal’s preferences [15].

Traditionally, however, GPs refer their patients to a specific provider based on, for instance, connections to that provider or their own medical judgement, instead of acquiring patients’ preferences regarding the place of treatment [11,16]. Consequently, patient choice hardly happens at the point of referral [3]. This is understandable, because referring a patient is a process that is heavily influenced by a complex of interrelated factors. For instance, little comparative information is available and GPs do not always trust the information that is available [17]. However, although it is not clear whether patient choice leads to better health outcomes than visiting the default, the introduction of patient choice of healthcare providers calls for changes to the referral process [16]. In accordance with policy on patient choice, GPs are expected to take on the task of supporting patients to be actively involved in their own care, including the choice of a healthcare provider. More specifically, they need, in their referrals, to incorporate patients’ preferences and to provide advice regarding the choice of a provider [3,16]. However, it is not clear if this policy is reflected in practice.

There is evidence that different factors influence GPs’ referral decisions, such as patient and GP characteristics [18]. Some GPs, for instance, are more positive towards facilitating patient choice than others [5] and some patients are more active regarding the choice of a provider than others [19]. The process that patients follow from their first demand for care until the end of their treatment, called the ‘healthcare path’, may have an additional influence on their referral pattern. Going to a hospital for treatment is not an isolated incident. Instead, visits to healthcare providers form a path of interconnected events. For example, a patient may go to the GP first, is then referred to a hospital for diagnostic purposes and, finally, is sent to another hospital department, or even another hospital, for treatment. While following this path, there may not be any clear opportunity to make a choice, particularly not independent of any previous care the patient has received [20-22].

### Research focus

We aim to investigate if policy regarding patient choice is reflected in practice at the point of referral, i.e. whether GPs help patients to make an active choice of a healthcare provider, for example by informing them about different referral options, giving them information about alternatives and asking for their preferences. Our research questions are: ‘at the point of referral, what is the role of the patient in choosing a healthcare provider?’; ‘to what extent does the GP influence the role that patients play in the referral decision?; and ‘to what extent do patients’ healthcare paths influence the role that patients play in the referral decision?’ Our study, with its demonstration of how patient choice is operating in practice at the point of referral, may provide guidance to GPs about how they can adjust their referral behaviour in order to comply with policy regarding patient choice and patients’ preferences for the provider they are referred to. Conversely, it may help policy makers understand how policy should be adapted to better match practice.

The study is conducted in the Netherlands. Here, patient choice of healthcare providers is encouraged since regulated competition was introduced during the healthcare reform of 2006 to enable patients to personalize their care and to improve healthcare quality and efficiency [4]. Citizens are obliged to take out healthcare insurance covering hospital costs incurred and GPs serve as gatekeepers to secondary care [23]. They are required to provide information about treatment alternatives in order to enable patients to play an active role in their own care [24].

## Method

### Recruitment of professionals

Video recordings were collected from GP-patient consultations as part of the ‘GP-patient communication study in 2007-2008’ [25]. GPs who participated in the study are all members of the Netherlands Information Network of General Practice (LINH), a representative network of 84 general practices and more than 330,000 patients [26]. A sample of 93 GPs was drawn from LINH of which 40 GPs (44%) from 20 practices agreed to participate in this study [25] (see [27] for the recruitment procedure). These 40 GPs were representative of Dutch GPs regarding gender and type of practice, but were, on average, 4 years older than the average Dutch GP. GPs were told that the study was about whether the healthcare reform in 2006 led to adjustments regarding the way they communicate with their patients. Consequently, they were unaware of the fact that our observations focused on referral decisions.

### Recruitment of patients and procedure

The 40 GPs agreed to have approximately twenty consecutive, standard consultations, videotaped. The recording with an unmanned digital camera took place on one or two random days, resulting in 808 consultations being recorded. A total of 77.6% of the patients agreed to participate. Those who refused were somewhat older, on average 48 years compared to 43 years, and were more often men.

All the GPs and patients who participated filled in an informed consent form before the recording of the consultation. Participants could withdraw their consent at any time, although no one did. Prior to the consultation, patients completed a questionnaire about their socio-demographic characteristics.

### Analyses

Where possible, two videotaped GP-patient consultations in which the patient was referred to a care provider were selected randomly per GP. The consultations were coded by one observer (JS) using an observation protocol we developed to describe the referral process (Additional file 1). We did not develop the observation protocol in advance, but based it on the random sample of the video recordings collected. We assumed when no new themes emerged that it covered the referral process and we considered it complete. When patients were referred for more than one condition then only the first condition mentioned was assessed for this study. The protocol consisted of 16 items, which address the following topics:

1. We used five items to observe the role that patients played in the choice of the healthcare provider they were referred to (items 1–5). Item 1 (with a three-point Likert scale) was used to observe the extent to which patients had input into the decision about where they were to be referred to (1 = little or no input of the patient, GP chooses referral; 2 = some input of the patient; 3 = a large amount of input of the patient, patient chooses referral). Patients’ scores on this item were based on their scores on the items 2–5. All of these items were equally important.

2. Six items were used to observe the extent to which GPs influence the role that patients played in the referral decision (items 6–11).

3. We developed four items that relate to different aspects of patients’ healthcare paths (items 12–15) in order to assess the role that patients’ healthcare paths played in the choice of the healthcare provider the patients were referred to. We coded whether patients visited that healthcare provider before, whether they previously visited another caregiver with their condition, whether they knew their diagnosis, or probable diagnosis, at the point of referral and the goal of their referral.

4. One open question (item 16) assessed the provider the patient was referred to.

To assess interrater reliability, a random ten per cent of the consultations were rated by a second observer independently (AV), resulting in sufficiently high average Kappa scores of 0.87 (range 0.38-1.00). The reliability of one item was below moderate (item 10) with a Kappa score of 0.38 [28], due to a different understanding of what constituted practical information (i.e. whether the name of the provider was practical information). After discussing this issue, this item was assessed by both AV and JS on all videos, and no conflicts were found comparing the results.

### Statistical analyses

The descriptive analyses and the interrater reliability calculation were performed using Stata 12. The explorative and qualitative nature of the data does not allow statistical analyses to be performed or for determining causation.

### Ethical considerations

The study was carried out according to Dutch privacy legislation. The privacy regulations were approved by the Dutch Data Protection Authority. Approval by a medical ethics committee was not required under Dutch law for this observational study [29]. Prior to consultation, written informed consent was obtained from all GPs and patients.

## Results

### Patient and GP characteristics

One GP did not refer any patients during the consultations recorded, five GPs referred only one patient, and one consultation was not eligible for inclusion because the patient’s attendant made the referral decision on his behalf. Ultimately, 72 videotaped consultations between 72 patients and 39 GPs were selected (55% of all consultations in which a referral took place).

Patients were referred to a large variety of healthcare providers. Most patients were referred to a physiotherapist (n = 13(18%)), followed by an orthopaedist (n = 12(17%)) and a cardiologist (n = 7(10%)).

Table 1 describes the characteristics of the patients and the GPs involved in the GP-patient consultations which were selected. The majority of both the GPs and the patients were male. Patients were on average 49.2 years old and GPs 51.2 years old. The majority of the patients had a medium education level.

**Table 1:** Background characteristics of the patients and the GPs per patient group

	**Total**	**No/little input**	**Some input**	**Much input**
	**(n = 72)**	**(n = 45)**	**(n = 9)**	**(n = 18)**
**GP**				
Age in years (M(SD))	51.2(6.0)	50.2(6.0)	51.4(5.4)	53.4(5.9)
Gender (n(%))				
Man	45(62.5)	29(64.4)	5(55.6)	11(61.1)
**Patient**				
Age in years (M(SD))^1^	49.2(21.9)	47.2 (21.7)	39.1(30.9)	58.4 (14.6)
Gender (n(%))				
Man	39(54.2)	27(60.0)	5(55.6)	7(38.9)
Education level (n(%))				
None	1(1.4)	0(0.0)	1(11.1)	0(0.0)
Low^2^	8(11.1)	5(11.1)	0(0.0)	3(16.7)
Medium^3^	30(41.7)	18(40.0)	2(22.2)	10(55.6)
High^4^	12(16.7)	8(17.8)	2(22.2)	2(11.1)
Missing	21(29.2)	14(31.1)	4(44.4)	3(16.7)
**Referral goal (n(%))**				
Diagnosis	43(59.7)	28(62.2)	7(77.8)	8(44.4)
Treatment	28(38.9)	16(35.6)	2(22.2)	10(55.6)
Second opinion	1(1.4)	1(2.2)	0(0.0)	0(0.0)

^1^Patients aged seventeen years or younger were also included in the calculation (n = 9). During these consultations, a parent was present who talked with the GP on behalf of the patient; ^2^Low = primary school or only vocational training; ^3^Medium = secondary school or intermediate vocational training; ^4^High = tertiary education.

### Role of the patient

At the point of referral, patients differed in the amount of input they had in their referral decision. Based on item 1, the patients were divided into three groups. The first group comprises patients who, at the point of referral by their GP, had little or no input into the choice of a healthcare provider (n = 45(63%)). The GP chose the referral option and patients simply agreed with the proposed option. For instance, a GP said: “I’d like to send you to that respiratory therapist who, originally, was a physiotherapist” and another GP chose a hospital on behalf of her patient because he stated that he did not have insight into the quality of hospitals. Most patients did not indicate a preference for a specific provider. The one patient who did was referred by his GP to another provider than the one he indicated a preference for. Nor did patients bring up a provider themselves and two of them (3%) asked their GP which provider they should visit, for instance “To which neurologist should I go?”.

The second group consists of patients who had a large amount of input into the choice of a referral option (n = 18(25%)). These patients chose the healthcare provider they were referred to themselves or asked for alternative options. For instance, one patient who needed arch supports said to his GP: “Listen, I’m getting a new corset in fourteen days and I saw that they make arch supports at [name orthopedist] as well”. Another patient indicated her preference for a consultant upon the following question from her GP: “who did you think of?” Patients indicated a preference for a specific provider (n = 13(72%)), often without the GP having to mention options, or asked for a preference (n = 9(50%)). Some of them explained why they preferred that provider, for instance “I can come with [name wife] then. Because [name wife] has an appointment with him at 11 a.m. too”(n = 6(33%)). Most patients brought up that provider themselves (n = 12(67%)). In the consultations in which the patient did not indicate a preference, the choice was left open and the patient had to choose a provider on their own after the consultation.

Patients from the third group fall in between the first and second group. This group consists of patients who were given a choice by their GP of a few options or told their GP that they did not want to be referred to a specific option (n = 9(13%)). For example, a GP said: “[name hospital A] or [name hospital B]?” Patients indicated a preference for a specific provider (n = 7(78%)), often when the GP mentioned a few options (n = 6(67%)), and a part of them explained why they preferred that provider (n = 4(44%)). However, they did not bring up a provider themselves. For instance, as one GP asked his patient “[name hospital A] or [name hospital B?]”, his patient replied with “I prefer [name hospital]. I’m fairly well known there”.

Table 1 shows the background characteristics of the patients per subgroup. Notably, the patients who were assigned to the ‘some input’ group deviate from the other two groups, most likely because of the small number of consultations in this group. In the remaining part of the results section, we leave the second group out of the reckoning when we focus on the different groups. The reasons are that the ‘some input’ patient group is very small and we want to be able to indicate the differences between consultations in which the patient has, or has not, input into the choice of a provider.

### Influence of the GP

The GP took the initiative to refer a patient to a healthcare provider in 56 (78% of all) consultations. For example: “I’d like you to go to the physiotherapist”. Notably, patients from the large amount of input group took the initiative for a referral in seven consultations (39%), while patients from the little or no input group took the initiative for a referral in five consultations (11%).

Concerning the question where to refer for treatment, the GP asked the patient whether they prefer a specific healthcare provider in 17 (24% of all) consultations. For instance, by saying “Do you prefer a specific hospital?” In the little or no input group, the GP asked for the patient’s preference in only 13% of the consultations. In these consultations the question was either a leading one or the patient indicated that he or she had no preferences, for instance, one GP said “I assume that you want to go to [name hospital]?” and one patient answered “I do not have insight into the quality of hospitals” when his GP asked for his preference. Additionally, in the majority of the consultations from the little or no input group, the GPs did not discuss alternative referral options (n = 40(89%)), but indicated a preference for a specific healthcare provider (n = 42(93%)). This was illustrated by the following quote: “You need to make an appointment [with the E.N.T. specialist] and I will give you a telephone number for that”. In most cases, (n = 41(91%)), GPs revealed this preference on their own initiative and not based upon the request of the patient. Furthermore, the GPs did not explain why they referred a patient to a specific provider (n = 34(76%)). They only provided an explanation in some cases, for instance saying: “Perhaps it is most useful if we ask him to look at you, so that you don’t have another doctor on your back”. In the large amount of input group, the GP asked for the patient’s preference in five consultations (28%). For instance by saying: “Who did you think of?”. Additionally, they indicated a preference for a specific healthcare provider in only three consultations (17%). In most of these cases, they explained why they referred the patient to that specific provider, for instance because “[name consultant] is a very calm and honest man” (n = 2(11%)).

GPs gave their patients information about healthcare providers in 43 (60% of all) consultations. However, in 28 (65%) of these consultations, this concerned solely practical information, such as opening times and the location of the institution. The GP only gave information about the quality of care, waiting times and the specialisation of the caregiver in a minority of the consultations (n = 9(13%), n = 5(7%) and n = 3(4%) respectively). Notably, in the large amount of input group, GPs always gave quality information in addition to practical information (n = 2(11%)). For instance: “Those are the two good ones. There are more physiotherapists in [location], where you live. You are free to choose”. In the little or no input group, the GP only gave quality information in some of the consultations (n = 4(9%)).

### The influence of patients’ healthcare paths

A small majority (n = 42(58%)) of the patients had already been to a healthcare provider before the consultation, either for diagnostic or treatment purposes. In addition, 27 patients (38%) had previously been to the specific provider to which they were referred. Furthermore, in half of the consultations (n = 36), the patient had already received a diagnosis or the GP gave the diagnosis or at least a probable one. Finally, in 43 (60%) of the consultations, the patient was referred for diagnostic, or further diagnostic, tests. Patients from the little or no input group were referred for diagnostic purposes instead of treatment in 28 consultations (62%), while patients from the a large amount of input group were referred for diagnostic purposes in eight consultations ((44%).

## Discussion

The majority of the patients had little input into the choice of a healthcare provider at the point of referral by their GP. Their GPs chose a healthcare provider for these patients. The GPs took the initiative for these referrals asking only for patients’ preferences in some of these consultations. They did not discuss alternative referral options. They indicated a preference for a specific healthcare provider but did not explain why. When they gave information it was solely practical.

Even though the majority of the patients had little input, a quarter still chose a healthcare provider themselves without the GP restricting their set of choices. These patients did not necessarily make a deliberate choice based on comparative information as is expected according to the policy on patient choice, but at least, they played a more active role in the choice of a provider at the point of referral. In the case of these patients, their GP seemed to have supported them in becoming actively involved in their choice of a healthcare provider. This indicates that differences exist in the roles that GPs and patients play in the choice of a healthcare provider at the point of referral. Patients’ healthcare paths seemed to have had some influence on the amount of input that patients had in their own referral. Patients who were referred for diagnostic purposes seem to have had less input into their choice of provider.

### Comparison with existing literature

Previous research found that GPs believe that a referral generally takes place upon a request by the patient [30]. We found, however, in the majority of the consultations, that the GP took the initiative to discuss a referral. The finding that most patients do not have much input into decisions taken at the point of referral is in line with other literature. According to existing research, patients often indicate that they visit the provider recommended by their GP [7-10] and GPs indicate that they choose a hospital on behalf of their patients [3]. Therefore, practice does not correspond to the idea that patients act as autonomous consumers. This can be explained by several facts. Firstly, some countries have a strong system of primary care gate keeping [11]. Secondly, in the Netherlands, a standard GP-patient consultation lasts only ten minutes, which may be a bit short for discussing different referral possibilities. Thirdly, patients may be reluctant to decide on a provider, amongst other factors because of their lack of insight into the quality of providers [13]. Finally, patients may not attach importance to the choice of a provider [31].

Although GPs’ major role in patients’ provider choices can be explained and there is no convincing research that proves that patient involvement in the referral decision leads to better health outcomes, by the fact that patient choice is encouraged GPs should not simply choose providers on behalf of their patient. Instead, according to legislation on patient choice, they are expected to incorporate patients’ views into their referrals, using a shared decision-making approach [16,32]. In addition to these laws, caregivers have always taken the responsibility for delivering good quality care and part of this is to inform patients about appropriate and high quality care in order to enable shared decision-making [33]. Research into other decisions related to care, such as the choice of a treatment, reveals that GPs should be able to identify patients’ preferences because almost all patients can express their priorities [34]. We found, however, that consistent with existing research [16], relatively few GPs support patients in becoming actively involved in their choice of a healthcare provider, for instance by informing patients about the quality of different referral options. Moreover, during the consultations that we observed, few GPs mentioned why they referred a patient to a specific healthcare provider. This finding is in line with existing literature, which found that GPs based their referrals on factors other than information on quality of care. Instead their opinion about a particular provider or connections to specific providers played a role [16,17].

Our results indicate that patients’ healthcare paths influence the role that patients play in the referral decision. Patients who are referred for diagnostic purposes instead of treatment seem to have had less input into the referral decision. This is in line with existing literature. Consistent with the ‘logic of care’, the nature of care might not be suited towards making an active choice of a provider [20,21]. Many situations erode the opportunity to make active choices, such as occasions when patients do not yet know their diagnosis at the point of referral. In that case, patients have to choose a hospital while being unaware of the care that they will need. It seems therefore natural that patients who are referred for diagnostic purposes are less inclined to make an active choice than patients who are referred for treatment.

### Strengths, limitations and further research

Existing research into referral decisions used focus groups, interviews or questionnaires to investigate GPs’ [3,5,16,18] and patients’ [9,10] roles in the choice of provider. Only a few studies exist that focused on actual referral data in order to investigate referral decisions [17,18]. These studies investigated the factors that influence GPs’ referral decisions, but provide no insight in what happens in the interaction between GP and patient while discussing the referral. We believe this is the first study that observed actual GP-patient consultations in order to analyse the role that patients play in the referral decision and whether GPs support patients in actively choosing a provider. Observations are a more objective source than self-reporting by GPs or patients, which could be biased. We were able to investigate what actually happened during the consultations by developing the observation protocol based on video recordings of actual GP-patient consultations. Another strength of this study is that the GPs participating are representative of Dutch GPs with regard to gender and the type of practice. It was also important that GPs did not know that our observations focused on referral decisions and that we analysed routine GP consultations instead of focussing on specific patient populations or relying on a trial situation. Therefore, our results represent the actual daily situation in general practice.

A limitation was that the video recordings were collected in 2007 and 2008. At that time, the Dutch healthcare system had only just been reformed, beginning in 2006. Therefore, the GPs might not have had enough time to adjust their practice accordingly. However, even today, GPs indicate that they choose a provider on behalf of their patients [3]. Therefore, there are no indications that practice has changed since 2007. A second limitation was that GPs and patients were not asked to fill out a questionnaire or participate in an interview investigating the referral process. Consequently, we were unaware of which provider patients ultimately visited, patients’ and GPs’ attitudes towards patient choice and what GPs and patients were thinking. For instance, a GP could have referred a patient to a particular provider because of its good reputation, without explaining this reason to the patient. That is, however, inherent in the nature of observational studies. Thirdly, the total number of consultations was insufficient to be able to compare referral patterns between GPs. Fourthly, the qualitative nature of the study and the fact that two consultations per GP were assessed did not allow statistical analysis to be performed nor for adjusting the results for differences between GPs or investigating if GPs adjust the way they communicate to the patient. Finally, although the reliability of one item was below moderate, we kept it in the observation protocol after addressing the cause of the below moderate reliability. Results regarding this question should be interpreted with some caution.

Future research could investigate whether the idea that GPs are expected to encourage patients to make an active choice of provider gains support from both GPs and patients. Research could also identify the barriers that can be encountered when GPs and patients take up their new roles and provide guidance on finding appropriate solutions for these barriers. Furthermore, it should be studied whether active choice leads to better health outcomes than visiting the default. Finally, future research should investigate why exactly patient choice hardly happens at the point of referral.

## Conclusion

GPs played a key role in choosing a healthcare provider in the majority of the GP-patient consultations. Often, the GP chose a healthcare provider on behalf of the patient. At the same time, however, policy on patient choice expects from patients that they make active choices of healthcare providers. It is assumed that this will ultimately lead to more personalized care of higher quality and efficiency. Our study shows, however, that the policy on patient choice is not reflected in daily practice. On the one hand, this could indicate that the policy needs adjustments. The current expectations for both GPs and patients regarding the choice of a provider may be unrealistic. On the other hand, to achieve the goals of the policy on patient choice, adjustments may be needed to practice. For instance, GPs could help patients more often to choose the provider that fits their needs and preferences. For example, they could investigate whether their patients want a more active role in the choice of a provider, identify their patients’ preferences regarding healthcare providers and discuss several referral options. Patients, for their part, could indicate their preferences and what they expect from their GP regarding the choice of a provider. Comparative information, accessible for both GPs and patients, and decision aids to support the GP assisting a patient making a choice of a provider might help in this matter. However, certain patients might not be interested in making active choices and would prefer to let their GP decide on a provider. Even then, GPs need to know patients’ preferences, because in a principal-agent relationship, it is necessary that the agent is fully informed about the principal’s preferences.

## Competing interests

The authors declare that they have no competing interests.

## Authors’ contributions

JN collected the video-recordings. JS, AV and JN developed the observation protocol. JS coded the videotaped consultations and AV and JS coded a random 10 per cent of the consultations. AV and JN drafted the manuscript and all other authors read and approved the final manuscript.

## Pre-publication history

The pre-publication history for this paper can be accessed here:

http://www.biomedcentral.com/1471-2296/14/189/prepub

## Supplementary Material

Additional file 1 Observation protocol. Click here for file
