# Supplementary material for: Are patients’ preferences regarding the place of treatment heard and addressed at the point of referral: an exploratory study based on observations of GP-patient consultations
Source: BMC Fam Pract. 2013 Dec 10;14:189. doi: 10.1186/1471-2296-14-189 (PMC4029442; doi:10.1186/1471-2296-14-189)
Supplement: Additional file 1 — Observation protocol. [file 1471-2296-14-189-S1.doc]

**Additional file 1. OBSERVATION PROTOCOL**

| GP code: Patient code: Observation date:  Consultation duration: Name observer: |
| --- |

1) How much input does the patient have regarding the choice of a care provider at the moment of referral?

1 2 3

□ NA

1 = little or no input. The GP chooses the provider and the patient simply agrees with the proposed institution or caregiver. It is obvious that the patient follows up the advice of the GP.

2 = some input. The patient is given a choice by the GP between a few providers or tells the GP that he or she does not want to be referred to a specific provider.

3 = a large amount of input. Not the GP, but the patient him or herself chooses the provider he or she is referred to or asked for alternative options. Alternatively, no decision is made during the consultation and the patient has to choose a care provider after the consultation.

2) Does the patient indicate a preference for a specific healthcare provider? □ Yes

a) the patient visits this provider frequently

b) the patient has been there before

c) the patient works at this provider

d) this provider has been recommended by family/friends/acquaintances

e) this provider had been recommended by the media (e.g. newspapers, internet)

f) it is unknown why the patient prefers this provider

g) it is the nearest provider

h) the patient knows the caregiver

i) this provider had been recommended by a patient organisation

j) this provider had been recommended by the patient’s insurer

k) other, i.e.: ……………………………

□ No

3) When does the patient indicate his or her preference for a specific provider?

□ After the GP mentioned referral options and/or information about these options

□ After the GP asked for this preference without having mentioned referral options

□ Without having been asked for this preference by the GP or without the GP having mentioned referral options and/or information about these options

□ NA

4) Who brought up the provider the patient is referred to?

□ GP

□ Patient

□ NA

5) For what reason or reasons does the patient want to be referred to a specific healthcare provider?

……………………………

□ NA

6) Does the GP ask the patient whether he or she prefers a specific healthcare provider?

□ Yes

□ No

7) Does the GP discus more than one referral option?

□ Yes

□ No

8) Does the GP indicate a preference for a specific healthcare provider?

□ Yes a) own initiative b) upon the patient’s request

□ No

□ NA

9) For what reason or reasons does the GP refer the patient to a specific healthcare provider?

……………………………

□ NA

10) Does the GP give the patient information about referral options?

□ Yes

⁯ a) Information about practical issues*

b) Information about the waiting list

c) Quality information

d) Information about the provider’s specialisation

□ No

* e.g. location, the way they act and opening times

11) Who takes the initiative to refer the patient to a healthcare provider?

□ GP

□ Patient

12) Has the patient already visited a healthcare provider prior to this consultation?

□ Yes

□ No

13) Has the patient already been diagnosed or does the GP give the diagnosis or probable diagnosis?

□ Yes

□ No

14) What is the goal of the referral?

□ Diagnostics

□ Treatment

□ Second opinion

15) Has the patient visited the provider to which he or she is referred in the past (healthcare institution or caregiver)?

□ Yes

□ No

□ Unknown

16) To which provider is the patient referred?

……………………………
